# Analysis of Heavy Metals in Foodstuffs and an Assessment of the Health Risks to the General Public via Consumption in Beijing, China

**DOI:** 10.3390/ijerph16060909

**Published:** 2019-03-13

**Authors:** Gang Liang, Wenwen Gong, Bingru Li, Jimin Zuo, Ligang Pan, Xinhui Liu

**Affiliations:** 1 Beijing Research Center for Agricultural Standards and Testing, Beijing Academy of Agriculture and Forestry Science, Beijing 100097, China; liangg@brcast.org.cn (G.L.); gongww@brcast.org.cn (W.G.); libr@brcast.org.cn (B.L.); 2 Risk Assessment Lab. for Agro-products (Beijing), Ministry of Agriculture, Beijing 100097, China; 3 Beijing Municipal Key Laboratory of Agriculture Environment Monitoring, Beijing 100097, China; 4 Station for popularizing agricultural technique, Daxing District, Beijing 100097, China; bjdxzuojimin@sohu.com; 5 Research Center for Eco-Environmental Engineering, Dongguan University of Technology, Dongguan 523808, China

**Keywords:** heavy metals, foodstuff, health risk, target hazard quotients, consumption

## Abstract

Consumption of foodstuffs is the most likely route for human exposure to heavy metals. This study was designed to investigate the toxic metals (cadmium (Cd), lead (Pb), chromium (Cr), arsenic (As), and mercury (Hg)) concentrations in different foodstuffs (cereals, vegetables, fruits, fish, and meat) and then estimate the potential health risks of toxic metals via consumption to the local residents in Beijing, China. Most of the selected toxic metal levels in the foodstuffs were lower than the maximum allowable concentrations of Pb, Cr, Cd, As, and Hg for Chinese foodstuffs recommended in the China National Food Safety Standard. The health risks associated with the toxic metals Pb, Cr, Cd, As, and Hg were assessed based on the target hazard quotients (THQs) proposed by the United States Environmental Protection Agency (US EPA). The THQ values of the foodstuffs varied and were 0.03–0.29 for Cr, 0.02–0.23 for Pb, 0.01–0.33 for Cd, 0.01–0.06 for As, and 0.00–0.04 for Hg, not exceeding the maximum level of 1. The total THQ (TTHQ) values were 0.88 for vegetables, 0.57 for cereals, 0.46 for meat, 0.32 for fish, and 0.07 for fruits. This indicates that the risk contribution from vegetable intake (38.8%) was significant in comparison to that from other foodstuffs. The TTHQ values were 0.96 for Cr, 0.54 for Pb, 0.50 for Cd, 0.19 for As, and 0.09 for Hg, suggesting that Cr was a major risk contributor (41.7%) for the local residents of Beijing, which should attract great attention. However, the THQ/TTHQ values were all below 1, suggesting no health risks to the local population through consumption. Furthermore, dietary weekly intakes (WIs) were also calculated and the values were all lower than the proposed limit of Provisional Tolerable Weekly Intakes (PTWI) established by the the Food and Agriculture Organization of the United Nations (FAO) and the World Health Organization (WHO). This suggests no additional health risks as well as consistency with the THQ results.

## 1. Introduction

Foodstuffs (such as vegetables, meat, cereals, fruits, and fish) are the most important part of the human diet, because they can provide the body with protein, vitamins, carbohydrates, calcium, iron, and other essential micronutrients (such as Cu, Zn) [1,2]. Therefore, the intake of various foodstuffs has become the main source of the nutrients, but also a route for the pollutants to enter into the human body [3]. Consequently, foodstuff safety has not only become an important food quality attribute, but also a major issue of public concern within the last decade [4,5].

As is known, heavy metals are a class of non-biodegradable pollutants, and they can accumulate and migrate in soil environments, which is the primary exposure route to humans/animals [6]. According to the data from the first national survey of soil contamination conducted by the Ministry of Environmental Protection and Ministry of Land and Resources of China, China faces serious soil metal pollution [7], and about 82.8% of the soil samples have exceeded the standard limit for heavy metals in soil [8]. Generally, industrial and municipal activity is considered as the main source of the land contamination of heavy metals, and wastewater irrigation in agricultural lands is the principal source of toxic metal contamination [6]. Once heavy metals are released into water and soil environments, they can accumulate in the food in the form of crops, vegetables, and plants [9,10]. Consumption of contaminated foodstuffs is the main way that heavy metals enter into the human body [11]. For example, more than 70% of dietary intake of cadmium is contributed via the food chain [12], so prolonged consumption of the crops/vegetables/plants grown on contaminated land may can lead to increased accumulation of heavy metals in the human body (such as in the liver and kidney) [13]. Some heavy metals, such as Cu, Zn, and Ni, act as micronutrients for the growth of human beings when present in trace quantities. However, some toxic heavy metals, such as Pb, Hg, Cr, Cd, and As (As is often classified into the heavy metal category due to similarities in chemical properties [14]), are hazardous to human health even at a trace level, especially for pregnant women and young children who are more vulnerable to toxic metal toxicity. Toxic metals can also cause disruption of the numerous biochemical processes in the human body, pose a serious health risk to humans [15], and eventually lead to an increased incidence of chronic diseases, such as neurological disorders, central nervous system destruction, deformity, and cancer [16,17,18,19]; for example, the intoxication of the toxic metal Cd can cause renal tubular dysfunction or anemia skeletal damage [17]. For this reason, concerns have been raised about the potential risks of toxic metal consumption in foodstuffs, and it is necessary to test toxic metal levels in the most frequently consumed foodstuffs to assess the risks to human health [20] and thus act to protect it.

The suburban and urban areas of Beijing, the capital of China, are polluted by various heavy metals from developing industrial operations and fast urban expansion [21]. However, information on the health risk assessment studies of toxic metals through consumption of foodstuffs is quite limited. Moreover, most of the previous studies have only focused on a single or a few kinds of foodstuffs [22,23,24]; thus, a comprehensive risk assessment of toxic metals from foodstuffs to the Beijing residents is urgently needed. Heavy metals like Cd, Cr, As, Hg, and Pb are the toxic metals that are most harmful to human health, and they are considered as the priority control pollutants by the United States Environmental Protection Agency (USEPA) [25]. Therefore, the purpose of this study was to estimate the health risks of toxic metals via consumption of foodstuffs to the general public of Beijing. The main objectives of this study were: (1) to determine the toxic metal (Cd, Cr, As, Hg, and Pb) concentration levels in 25 different kinds of foodstuffs; (2) to assess the potential health risks posed by these foodstuffs.

## 2. Materials and Methods

### 2.1. Sample Collection

The mostly consumed foodstuffs, including fish (hairtail, white amur, carp, shrimp, crucian), vegetables (cauliflower, white gourd, cabbage, eggplant, potato, cucumber, carrot, haricot bean, onion), cereals (millet, flour, corn, rice), meat (mutton, beef, chicken, pork) and fruits (pear, apple, banana) were collected in Beijing, China. The samples were collected in four different sites and three replicates for each foodstuff were collected in each site.

### 2.2. Sample Preparation and Analytical Methods

According to a previous study [2], the collected vegetables and fruits were firstly washed three times with de-ionized water before being cut into slices, and dried until they reached a constant weight. The dried samples were then ground in a porcelain mortar and passed through a 200-mesh sieve and finally stored in polyethylene bags. Fish samples were frozen at −20 °C until dissection. Then, the muscle of the fish samples was removed with a plastic knife, homogenized, weighed, and dried. A similar pretreat method was applied for the meat samples. The samples of cereals were firstly washed with de-ionized water and then dried to a constant weight prior to use.

A 0.2 g piece of the as-prepared sample was accurately weighed and transferred to a screw-cap polytetrafluoroethylene digestion vessel. A mixture of 3 mL nitric acid and 30% hydrogen peroxide (V:V = 2:1) was added and then left to predigest for 12 h at room temperature. The vessels were then sealed and placed in an oven at 160 °C for further acid digestion. Once the digestion process was completed, the vessels were allowed to cool to room temperature. The digested solution was diluted to 10 mL with Milli-Q water, and ready for inductively coupled plasma-atomic emission spectrometry (ICP-AES) analysis [26]. For As and Hg analysis, 1 mL of the diluted solution was transferred to a 5 mL vessel, then 1 mL thiocarbamide (5%) and 3 mL nitric acid (10%) were respectively added and then left to react for 5 h at room temperature, to ready them for atomic fluorescence spectrometry (AFS) analysis [27].

### 2.3. Instrument Measurements

The certified standard solutions of Cd, Cr, Pb, As, and Hg (1 mg/mL) were purchased from the National Institute of Metrology, China. The working standard solutions were prepared from the standard solutions with Milli-Q water (18.2 MΩ cm resistivity) from a Millipore Milli-Q system (Thermo Scientific EASYpure II, Waltham, MA, USA). Calibration was performed by analyzing the prepared working standard solutions and two agent blank samples. Concentrations of Cd, Cr, and Pb were determined using an Inductively Coupled Plasma-Atomic Emission Spectrometry instrument (SPECTRO Analytical Instruments GmbH, Kleve, Germany) with axial viewing configuration. Total concentrations of As and Hg were determined using Atomic Fluorescence Spectrometry (AFS-930) (Beijing Jitian instrument Ltd., Beijing, China).

### 2.4. Quality Assurance and Control

The reagent blank samples, which were used for correcting the instrument readings, were digested in the same way as the foodstuff samples. The method limits of detection (MLOD) was calculated based on three times the standard deviation for digestion blanks, and the MLODs were 0.01, 0.05, and 0.12 mg/kg for Cd, Cr, and Pb, and 0.40, and 0.25 μg/kg for As, and Hg, respectively. The accuracy of the methods was validated by three replicate measurements. Furthermore, a sample with standard metal concentration was then measured to evaluate the instrumental accuracy after twenty foodstuff samples.

All glassware and plasticware were pre-washed three times with de-ionized water, and then soaked in HNO_3_ solution (30%, v/v) overnight. After soaking, the glassware and plasticware were further rinsed three times with de-ionized water and then dried in an oven.

### 2.5. Methodology for Health Risk Assessment

The method for health risk assessment is based on non-carcinogenic effects, and the risk is expressed as a target hazard quotients (THQ). The methodology for estimating THQ is described in detail by the US EPA [28]. A THQ value of less than 1 means that the exposure level is smaller than the reference dose, which assumes that the daily exposure at this level is unlikely to cause any obviously deleterious effects to the exposure population. That is, a THQ below 1 means the adverse effects are negligible. If the value of THQ exceeds 1, then there is a chance that non-carcinogenic effects may occur, with a probability which tends to increase as the value of THQ increases. The models for estimating THQs are [29]:

(1) $$THQ=\frac{E_{F}E_{D}F_{IR}C}{R_{FD}W_{AB}T_{A}}\times 10^{-3}$$

(2) ΣTHQ=THQ

where *E_F_* is exposure frequency (365 days/year); *E_D_* is the exposure duration (70 years), equivalent to the average lifetime [30]; *F_IR_* is the food ingestion rate (g/person/day); *C* is the metal concentration in foodstuffs (μg/g); *R_FD_* is the oral reference dose (mg/kg/day); *W_AB_* is the average body weight (60 kg for adults) [31]; and *T_A_* is the averaging exposure time for non-carcinogens (365 days/year × number of exposure years, assuming 70 years in this study) [32].

## 3. Results and Discussion

### 3.1. Heavy Metal Concentrations in Different Foodstuffs

The average concentrations of the toxic metals Cd, Cr, Pb, As, and Hg, along with the relevant standard deviation values are listed in Table 1. As shown in Table 1, there was a striking difference in the concentration for the studied toxic metals in different foodstuffs. According to the China National Food Safety Standard [33], the average concentrations of the concerned toxic metals in most of the selected foodstuffs were lower than the maximum limit of normal values. However, the concentrations of Pb in chicken, fish, white gourd, pork, and the Hg concentrations in millet and mutton were relatively higher than the limit standards of Pb (0.2 mg/kg) and Hg (0.02 mg/kg) in food. Therefore, there is need to estimate the health risks of heavy metals via consumption to the general public of Beijing.

### 3.2. Concentration of Heavy Metals in Different Foodstuff Groups

**Figure 1:**
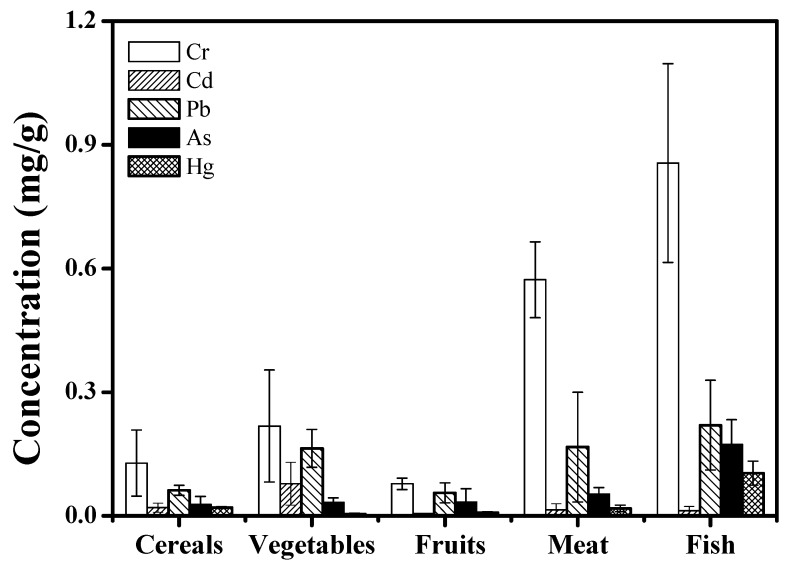
Mean metal concentrations of different species (mg/kg) (dry weight).

To better reflect the heavy metal concentrations in foodstuffs, the selected 25 types of foodstuffs were classified to five foodstuff groups (fruits, meat, vegetables, fish, and cereals). The mean and varying range of individual heavy metal concentrations in cereals, fruits, vegetables, meat, and fish were calculated and presented in Figure 1.

As shown in Figure 1, the average concentrations of Cr, Cd, Pb, As, and Hg were 0.128, 0.020, 0.062, 0.028, 0.020 mg/kg in cereals; 0.218, 0.078, 0.164, 0.033, 0.005 mg/kg in vegetables; 0.078, 0.006, 0.056, 0.034, 0.008 mg/kg in fruit; 0.573, 0.015, 0.167, 0.053, 0.018 mg/kg in meat; and 0.856, 0.013, 0.220, 0.174, 0.104 mg/kg in fish, respectively. Similarly, there was also a striking difference in heavy metal levels in different foodstuff groups; generally Cr presented in the highest concentration, and Cd and Hg presented in the lowest. Interestingly, the same decreased order of the examined heavy metals were observed for cereals, fruits, meat, and fish, i.e., Cr > Pb > As > Hg > Cd. A slightly different order occurred in the case of vegetables: Cr > Pb > Cd > As > Hg. However, the average values were all lower than the maximum allowable concentration of consumed foodstuffs in China. Thus, the food consumed is not a potential toxic source of the analyzed heavy metals for local inhabitants.

### 3.3. Heavy Metal Contribution of the Foodstuff Groups

Heavy metals can enter into human bodies and accumulate in the body, reaching toxic concentrations, i.e., higher than acceptable limits [34]. Therefore, the estimated total intakes corresponding to the different elements through food consumption were calculated and listed in Table 2. Dietary daily intakes for the examined heavy metals was calculated by multiplying the respective concentration in each kind of foodstuff by the weight of that foodstuff group consumed by an average individual [35]. For inhabitants of the studied areas, the daily consumption rates of foodstuffs were 366 g/person/day for cereals, 252 g/person/day for vegetables, 69 g/person/day for fruits, 105 g/person/day for meat, and 45 g/person/day for fish [36]. As shown in Table 2, the estimated daily intakes of heavy metals through food consumption decreased in the following order: Cr > Pb > As > Cd > Hg and were 205.52 μg/d for Cr, 95.15 μg/d for Pb, 34.02 μg/d for As, 29.48 μg/d for Cd, and 15.63 μg/d for Hg.

**Figure 2:**
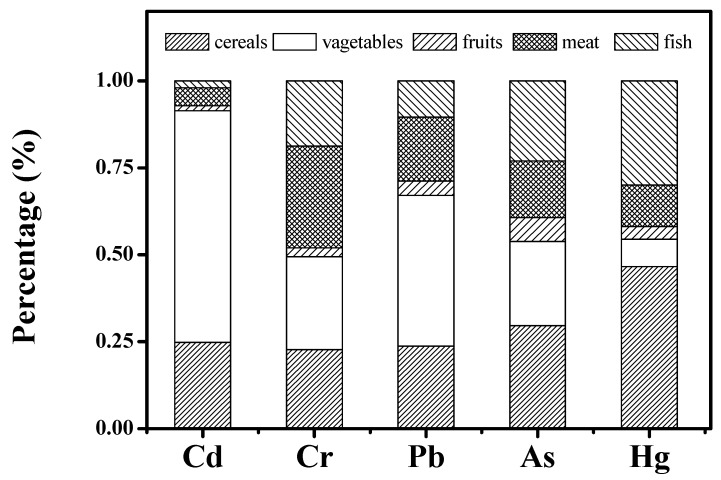
Percentage contribution analysis of different heavy metals via consumption.

Furthermore, to better analyze the source difference of heavy metals from foodstuff groups, we compared the percentage contribution to the heavy metals from different foodstuff groups. As presented in Figure 2, the percentage contribution for the foodstuff groups varied greatly among different heavy metals. The major two distributors for each heavy metal were vegetables (66.7%) and cereals (24.8%) for Cd; cereals (46.6%) and fish (26.7%) for Hg; vegetables (43.3%) and cereals (23.8%) for Pb; cereals (29.6%) and vegetables (24.2%) for As; and meat (29.3%) and vegetables (26.7%) for Cr. The above results show that vegetables contributed the most to the Cd and Pb dietary intake, and cereals contributed the most to the Hg and As dietary intake. More specifically, the consumption of vegetables and cereals accounted for 91.5% of total Cd intake, 67.1% of total Pb intake, and 53.8% of total As intake. Therefore, it can be inferred that vegetables and cereals are the two major sources of heavy metals that enter human body via consumption. Consequently, the heavy metal levels in vegetables and cereals should paid special attention and the government supervision should be enhanced to decrease the potential health risks of heavy metals to humans via consumption. Although the highest concentration of Hg occurred in fish, the consumption of cereals is the main source of Hg dietary intake in our study, which is not consistent with the previous studies. This might be attributed to the diet of local inhabitants, nevertheless the health risks of Hg via consumption of cereals should be focused on in the future.

### 3.4. Health Risk Assessment of Heavy Metals via Food Consumption

To evaluate the health risk of heavy metals via food consumption to the local inhabitants, the weekly intakes of the toxic metals were analyzed and compared with the provisional tolerable weekly intakes (PTWIs) recommended by the Food and Agriculture Organization of the United Nations (FAO) and World Health Organization (WHO). As shown in Table 3, the calculated weekly intakes (WIs) in this work were calculated to be 24.0 for Cr, 11.1 for Pb, 4.0 for As, 1.8 for Hg, and 3.4 for Cd (μg/kg bw/week), which were all appreciably below the respective PTWIs [37,38], indicating that these intake levels do not pose a health concern for the inhabitants.

Furthermore, THQs of studied metals through consumption of foodstuffs by the Beijing local inhabitants were derived based on the methodology described in the US EPA used for assessing health risks. Since the foodstuffs (vegetables, cereals, meat, fruits, and fish) were not locally produced, the averaged concentrations of heavy metals were used for calculation of THQs for the Beijing residents in this study. The THQs are listed in Table 4.

As shown in Table 4, the THQ values varied from 0.03–0.29 for Cr, 0.02–0.23 for Pb, 0.01–0.33 for Cd, 0.01–0.06 for As, and 0.00–0.04 for Hg. The THQ values of the studied metals were all much lower than 1, especially for the THQ values of As and Hg, suggesting that the health risks associated with heavy metal exposure is not significant. The total THQ (TTHQ) for Cr, Pb, Cd, As, and Hg due to consumption were 0.96, 0.54, 0.50, 0.19, and 0.09, respectively. The TTHQ values were generally less than 1, only the TTHQ for Cr was close 1. This suggests that the Beijing area does not face a significant potential health risk caused by the intake of a single metal through the consumption of all the studied foodstuffs. The relative contributions of Cr, Pb, Cd, As, and Hg to the TTHQ from all foodstuff consumption were also calculated. Cr is a major risk contributor for the residents in Beijing, accounting for 41.7% of the total TTHQ, while the risk contribution from As and Hg is relatively low, accounting only for 8.3% and 3.9%, respectively. In addition, the TTHQ (sum of individual metal THQs via consumption for each of the vegetables, cereals, meat, fruit, and fish) were also calculated. The values were 0.88, 0.57, 0.46, 0.32, 0.07, respectively, which in each case were lower than 1 as well, indicating that the Beijing residents are not exposed to harmful health effects. The TTHQ value for vegetables was 0.88, which is higher than those of cereals, meat, fruits, and fish for the Beijing inhabitants. This indicates that the potential health risk is mainly due to the intake of vegetables in the Beijing area (38.2%), while that from fruits only contributes a minor fraction (3.0%). The above results also confirmed that there is no potential health risks via consumption of vegetables, cereals, meat, fruits, and fish alone. 

However, the TTHQ values reached 2.30 when all metal intake via vegetables, cereals, meat, fruits, and fish were combined. This indicates that the local inhabitants living in the Beijing area were exposed to possible adverse health effects. It should also be noted that the THQ value is a highly conservative and relative index and the TTHQ value exceeding 1 does not actually mean that local people have been exposed to adverse health effects [3]. However, as demonstrated in our study, the potential health risk of Cr and the risk through intake of vegetables are the highest. Therefore, it is necessary to strengthen the monitoring of Cr levels in foodstuffs and also a consumption advisory note should be issued.

## 4. Conclusions

The levels of toxic metals (Cd, Cr, Pb, Hg, and As) in foodstuffs were analyzed and their potential health risks were estimated. The results show that the levels of the concerned toxic metals in most of the foodstuffs were lower than the maximum limit of normal values recommended by the China National Food Safety Standard, except for Pb concentrations in chicken, fish, white gourd, and pork, and Hg concentrations in millet and mutton. While the dietary WIs and THQ analysis results indicate no additional health risks associated with toxic metals to the local population via consumption. Therefore, from the health risk assessment of toxic metals, we can conclude that the daily foodstuffs of vegetables, cereals, meat, fruits, and fish are safe to eat for the local population. However, there are many routes for the migration of heavy metals into humans, such as food, water, air, so the potential health risks might be underestimated. In addition, although the biological indicators have been widely applied for the monitoring of environmental pollutants in health exposure risk assessments, the relationship between external and internal levels of pollutants has not yet been adequately established. Therefore, it is difficult to accurately assess the potential health risks associated with toxic metals.

## Figures and Tables

**Table 1:** Heavy metal concentrations in different foodstuff types (mg/kg).

Foodstuff Kinds	Cr	Cd	Pb	As	Hg
crucian carp	0.709 ± 0.169	0.009 ± 0.004	0.227 ± 0.122	0.079 ± 0.024	0.094 ± 0.024
carp	0.867 ± 0.412	0.004 ± 0.002	0.036 ± 0.189	0.224 ± 0.280	0.089 ± 0.019
white amur	0.769 ± 0.169	0.009 ± 0.001	0.322 ± 0.174	0.196 ± 0.173	0.097 ± 0.052
hairtail	0.667 ± 0.111	0.029 ± 0.004	0.276 ± 0.168	0.156 ± 0.038	0.084 ± 0.009
shrimp	1.266 ± 0.376	0.013 ± 0.010	0.240 ± 0.097	0.216 ± 0.068	0.156 ± 0.045
carrot	0.242 ± 0.039	0.134 ± 0.074	0.194 ± 0.058	0.040 ± 0.009	0.007 ± 0.002
haricot bean	0.372 ± 0.096	0.011 ± 0.012	0.187 ± 0.015	0.037 ± 0.010	0.006 ± 0.004
eggplant	0.022 ± 0.036	0.142 ± 0.098	0.077 ± 0.038	0.040 ± 0.015	0.005 ± 0.003
cauliflower	0.379 ± 0.046	0.043 ± 0.010	0.182 ± 0.154	0.022 ± 0.011	0.005 ± 0.001
cucumber	0.219 ± 0.058	0.010 ± 0.008	0.192 ± 0.045	0.022 ± 0.011	0.005 ± 0.003
onion	0.036 ± 0.010	0.040 ± 0.015	0.155 ± 0.036	0.052 ± 0.031	0.004 ± 0.003
cabbage	0.216 ± 0.182	0.104 ± 0.030	0.138 ± 0.194	0.030 ± 0.016	0.003 ± 0.003
potato	0.351 ± 0.205	0.101 ± 0.097	0.121 ± 0.048	0.018 ± 0.003	0.008 ± 0.002
white gourd	0.122 ± 0.052	0.116 ± 0.034	0.227 ± 0.100	0.033 ± 0.006	0.002 ± 0.002
onion	0.143 ± 0.104	0.027 ± 0.015	0.070 ± 0.029	0.053 ± 0.022	0.019 ± 0.010
millet	0.057 ± 0.006	0.027 ± 0.005	0.072 ± 0.018	0.026 ± 0.007	0.023 ± 0.006
corn flour	0.076 ± 0.050	0.003 ± 0.002	0.060 ± 0.028	0.007 ± 0.002	0.018 ± 0.013
wheat flour	0.234 ± 0.038	0.023 ± 0.007	0.045 ± 0.017	0.024 ± 0.007	0.020 ± 0.014
pork	0.483 ± 0.046	0.003 ± 0.003	0.029 ± 0.049	0.043 ± 0.009	0.015 ± 0.005
beef	0.504 ± 0.056	0.015 ± 0.008	0.201 ± 0.111	0.077 ± 0.047	0.010 ± 0.004
mutton	0.654 ± 0.024	0.010 ± 0.004	0.128 ± 0.012	0.046 ± 0.003	0.029 ± 0.018
chicken	0.650 ± 0.125	0.031 ± 0.041	0.291 ± 0.117	0.045 ± 0.009	0.017 ± 0.001
apple	0.085 ± 0.078	0.005 ± 0.003	0.080 ± 0.035	0.050 ± 0.003	0.009 ± 0.001
pear	0.061 ± 0.128	0.007 ± 0.002	0.032 ± 0.019	0.022 ± 0.014	0.010 ± 0.001
banana	0.087 ± 0.123	0.005 ± 0.004	0.056 ± 0.014	0.010 ± 0.002	0.006 ± 0.003

Cr-chromium, Cd-Cadmium, Pb-lead, As-Arsenic, Hg-Mercury.

**Table 2:** Estimated dietary intake of metals via food consumption (μg/d).

Metal	Cereals	Vegetable	Fruit	Meat	Fish	Total Intake
Cd	7.32	19.63	0.41	1.54	0.58	29.48
Cr	46.67	54.85	5.36	60.14	38.50	205.52
Pb	22.60	41.24	3.86	17.54	9.91	95.15
As	10.07	8.23	2.35	5.54	7.84	34.02
Hg	7.27	1.23	0.58	1.86	4.68	15.63

**Table 3:** Comparison of weekly intakes (WIs) with provisional tolerable weekly intakes (PTWIs) (μg/kg bw/week).

	Cd	Cr	Pb	As	Hg
Calculated WIs	3.4	24.0	11.1	4.0	1.8
PTWIs **	7	1050	25	15	5

** PTWI [37,38] reported in the literature (μg/kg bw/week).

**Table 4:** Target hazard quotients (THQ) and total target hazard quotients (TTHQ) of the studied metals caused by the consumption of foodstuffs for inhabitants in Beijing.

	THQ					TTHQ
	Cr	Pb	Cd	As	Hg	
Vegetables	0.26	0.23	0.33	0.05	0.01	0.88
Cereals	0.22	0.13	0.12	0.06	0.04	0.57
Meat	0.29	0.10	0.03	0.03	0.01	0.46
Fish	0.18	0.06	0.01	0.04	0.03	0.32
Fruits	0.03	0.02	0.01	0.01	0.00	0.07
TTHQ	0.96	0.54	0.50	0.19	0.09	2.30
